# Modular Ti-6Al-4V system for *in vitro* optimization of implant materials

**DOI:** 10.3389/fbioe.2025.1661278

**Published:** 2025-09-29

**Authors:** Charlotte von Heckel, Heike Walles, Georg Hasemann, Manja Krüger

**Affiliations:** ^1^ Department of Process and Systems Engineering, Core Facility Tissue Engineering, Institute of Chemistry, Otto-von-Guericke University, Magdeburg, Germany; ^2^ Department of Mechanical Engineering, Institute of Materials, Technologies and Mechanics, Otto-von-Guericke University, Magdeburg, Germany

**Keywords:** biomaterials, titanium, titanium alloy, implant, porosity

## Abstract

**Introduction:**

Titanium is a widely used biomaterial for implants. Research is focused on optimizing titanium by modifying its surface structure and porosity to prevent complications such as implant loosening or foreign body reactions. However, changes to implant materials necessitates preclinical testing, often involving animal testing, which gives rise to ethical concerns. This underscores the significant demand for test systems that can reduce time and costs. Although *in vitro* models can provide valuable initial insights into the biological evaluation of biomaterials and help reduce the need for animal experiments, dedicated test systems for biomaterial evaluation remain limited.

**Methods:**

In our work, we developed modular 3D porous stacked models using Ti-6Al-4V sheets with different structural designs. These models are used to demonstrate the migration of primary human fibroblasts into the implant through fluorescence microscopy and investigate the effects of pore size. In addition, Ti alloy sheets with slots were polished and ground (1,200 grid SiC) to examine differences in cell adhesion and migration for different surface properties.

**Results:**

The MTT results indicate similar cell growth on the different patterns and surface conditions, which suggests the use of more biomimetic structures in the future.

**Discussion:**

Consequently, our model serves as a screening system for optimization of porosity and surface conditions of implant materials, which contributes to early-stage *in vitro* biocompatibility and cell migration studies.

## 1 Introduction

Metallic biomaterials such as ASTM-graded pure titanium (Ti), Ti alloys, 316 L stainless steel, and Co-based alloy (ASTM F75) demonstrate very good biocompatibility and mechanical properties and are thus being used as commercially available implant materials (ASTM F75–07, 2008). Among those, Ti-Al-V (Kuroda et al., 1998; Sidambe, 2014; Ali et al., 2024; Moon et al., 2024), Ti-Mo-Zr-Al (Kim et al., 2000), Co-Cr-Mo (Crackau et al., 2020; Döring et al., 2019), and porous Ta (Han et al., 2019) implants possess also good bio- and cell compatibility (Kuroda et al., 1998) and are used in several implant applications, i.e., as hip, shoulder, or dental implants.

Titanium and its alloys are widely used as biomaterials for orthopedic implants, given their corrosion resistance and biocompatibility (Gouda et al., 2021). Titanium alloys, especially Ti-6Al-4V, are used as the main metallic biomaterials for cementless implants. In 2022, Ti-4Al-6V was the most commonly used biomaterial in 77% of all hip replacement cases in Germany according to the Arthroplasty Register (Grimberg et al., 2023). Thus, Ti-6Al-4V has become a gold standard in hip arthroplasty. However, one major disadvantage is the aseptic loosening of the implant due to weak interactions between the implant surface and the surrounding tissue. This can induce infections as well as foreign body reactions, such as inflammation (Noskovicova et al., 2021). It may also lead to fibroblasts causing fibrous encapsulation of the implant by forming collagen around it (Kastellorizios et al., 2015). The encapsulation might result in reduced function or implant loosening (Welch et al., 2020; He et al., 2017). In order to overcome these problems, the characteristics of the implant can be optimized, including surface properties and porosity (Bandyopadhyay et al., 2010). Porosity has also an influence on the elastic modulus of the implant. This is important since implants are often too stiff, resulting in a stress-shielding effect. An optimized porosity should enhance bone growth into the implant, which has a positive impact on the stability and biocompatibility (Ødegaard et al., 2021).

In addition to its effect on mechanical properties, porosity—encompassing parameters such as pore size, geometry, and interconnectivity—exerts a significant influence on cell behavior (Mukasheva et al., 2024). Research has demonstrated that the presence of small pores has a positive effect on cell adhesion, primarily due to their ability to provide stronger initial support. However, the cell growth potential is restricted owing to the available space (Jiao et al., 2023; Han et al., 2021). Consequently, a minimum pore size of 100 µm is generally recommended (Karageorgiou and Kaplan, 2005; Zadpoor, 2015). In contrast, larger pores offer greater space for cell proliferation and migration, as well as enhanced oxygen and nutrient supply (Mukasheva et al., 2024). However, markedly high pore size can have a detrimental effect on cell anchoring and migration (Jiao et al., 2023). The optimal pore size remains a subject of ambiguity within the current literature. Han et al. found that a pore size of 200 µm was optimal for bone marrow mesenchymal stem cell (BMSC) growth (Han et al., 2021). In the relevant literature on bone tissue regeneration, the range of typical pore sizes is summarized as up to 700 µm by Mukasheva et al. (2024). In a study conducted by Markel et al., the adhesion and proliferation of fibroblasts were examined on Ti cylinders of varying pore sizes. The findings of this research suggest that fibroblasts exhibit enhanced growth and viability within larger pore sizes (measuring 700 and 1,000 mm, respectively) (Markel et al., 2022). Beyond the impact of pore size, pore geometry also has been demonstrated to influence bone tissue regeneration, a topic that has been reviewed by Zadpoor (2015). It has been demonstrated that variations in the curvature can influence cell adhesion, cell migration, and cell morphology. Research has demonstrated that concave surfaces yield superior outcomes in comparison to convex surfaces. It was proposed that these parameters have a role in the stimulation and guidance of cells.

Further focus of current research is on surface properties of implant materials as they play a key role in tissue integration. Rough surfaces have been shown to promote bone integration, while smoother surfaces are beneficial for soft tissue integration (Rabel et al., 2021). Furthermore, surface patterning approaches are being extensively explored to modulate cell behavior and prevent bacterial adhesion, thereby reducing the risk of implant-associated infections (Regenberg et al., 2021; Okulov et al., 2020; Watanabe et al., 2023).

Furthermore, Al and V were dissolved from the Ti-base alloy within the body tissues, thus being a cause for concern. Al may act as a growth inhibitor for bones and is a possible cause of Alzheimer’s disease (Eliaz, 2019; Kandimalla et al., 2016). Vanadium is known to have strong cytotoxicity. Thus, V-free Ti-based alloys such as Ti-6Al-7Nb, Ti-5Al-13Ta, and Ti-13Zr-13Nb are under development and investigation (Semlitsch et al., 1985; Semlitsch et al., 1992; Goodman et al., 1993).

Recent developments have addressed refractory metal-based materials, especially refractory high-entropy alloys (RHEA) based on TiZrNbTaMo, TiZrHfNbTaMo, or TiZrHfCrMo (Wang and Xu, 2017; Iijima et al., 2021; Nagase et al., 2020). RHEAS provide good bio- and cell compatibility, as shown for fibroblasts and osteoblasts (Iijima et al., 2021; Nagase et al., 2020), and endow comparable mechanical properties as compared to those of Ti-6Al-4V, by slightly lower values of Young’s modulus, which would be favorable in terms of stress shielding.

The development of new implant materials and surfaces has become a major field of research on bio-medical materials. However, prior to their introduction into the market, preclinical and clinical studies are mandatory to determine their safety. These requirements are consistent with international standards, such as ISO 10993, which provides a framework for biological evaluation of medical devices (DIN EN ISO 10993-1:2021-05, 2021). These studies usually involve animal testing, which makes the process costly, time-consuming, and ethically controversial. However, certain biological responses can be evaluated through *in vitro* testing, such as the assessment of cytotoxicity (ISO 10993-5), for instance, helping reduce the need for animal experiments in this field and thereby aligning with the 3R principle. Nevertheless, there remains a need for the development of test systems that are specifically designed for biomaterials (Frisch et al., 2023).

To address these concerns, we aim to develop a modular system that enables different porosity and surface structures through defined combinations. These structures can be colonized with human cells to study biocompatibility and cell migration. The aim of this study is to establish the system as a screening platform for assessing scaffold performance in terms of cell behavior and tissue interaction. The influence of surface topography on primary human cell growth and behavior on Ti-6Al-4V substrates is investigated, specifically by comparing the effects of ground and polished surfaces. This contributes to the development of optimized biomaterials for clinical use.

## 2 Materials and methods

### 2.1 Preparation of Ti-6Al-4V samples

As a substrate material, purchased Ti-6Al-4V sheets (Titan-Metalle Deutschland GmbH, Grade 5, DIN 3.7165, ASTM B265) were cut via an industrial laser Trumpf TruLaser Cell 7020 with a dimension of 26 × 20 × 2.5 mm to fit in a standard 6-well plate. Slot patterns of varying designs were cut into the platelets, either oriented horizontally (H), vertically (V), or in rectangles (R) (Table 1) to vary the pore geometries via different stacking sequences. Horizontal and vertical slots have a width and distance of 1 mm, while the rectangular slots have an edge length of 2 mm in a distance of 1 mm. The Ti alloy sheets were either manually ground with 1200 grid SiC paper or polished to prepare different surface conditions. The platelets were designed to mimic different geometrically distinct porosities to study cell migration. Therefore, different stacking combinations were chosen (Table 1), while cells were initially seeded exclusively on the top platelet. The samples were placed on top of each other without leaving any gap between them. As illustrated in the top view images of the stacked models, it is demonstrated that pore channels exhibit dimensions of up to 1 mm. A cross-section of the pores reveals that potential migration pathways exhibit slight variations in the sequence.

**TABLE 1:** Visualization of Ti alloy sheets with different patterned slots and their properties. The combinations describe the order of the stack from top to bottom. Pore channels and potential migration paths are illustrated through top and cross-sectional view.

Platelets	Horizontal, 'H'	Vertical, 'V'	Rectangular, 'R'
	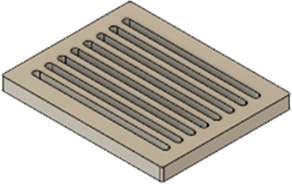	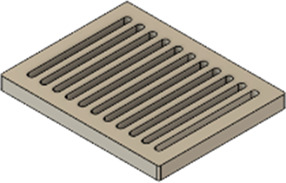	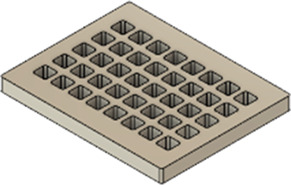
Surface area	1,537 mm^2^	1,561 mm^2^	1,464 mm^2^
Porosity	33%	38%	33%
Combinations	‘HVR'	‘VRH'	‘RHV'
Top view of the stacked model	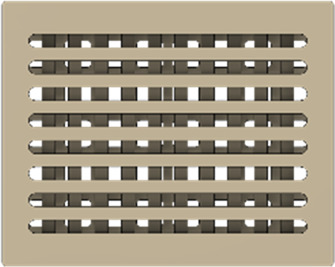	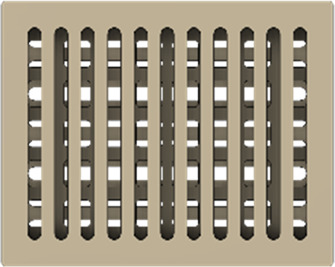	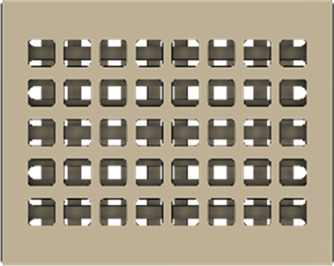
Cross-sectional view of potential migration paths	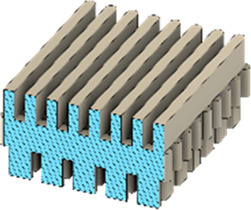	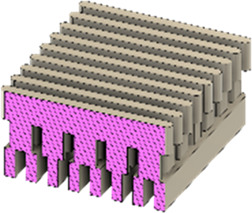	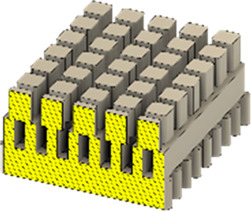

### 2.2 Cell culture

Primary fibroblasts were isolated from fascia biopsies under the approval of the Local Ethics Committee of the University of Wuerzburg (182/10) and informed consent of the patients. Cell expansion was performed in the fibroblast medium, consisting of Dulbecco’s modified Eagle’s medium (DMEM|High Glucose, Sigma-Aldrich D5796) supplemented with 10% fetal calf serum (FCS, Bio&Sell FBS.S0615) (Pudlas et al., 2011). The cells were cultivated in a humidified CO_2_ incubator (5% CO_2_, 37 °C) (ThermoFisher BBD 6220).

All Ti-6Al-4V samples were sterilized prior to use as stated in 2.3. According to the combinations listed in Table 1, the bottom and middle plates were transferred using a sterile forceps to a six-well plate, covered with 3 mL of the fibroblast medium and placed in an incubator. The top platelets were placed in a Petri dish.

**FIGURE 1:**
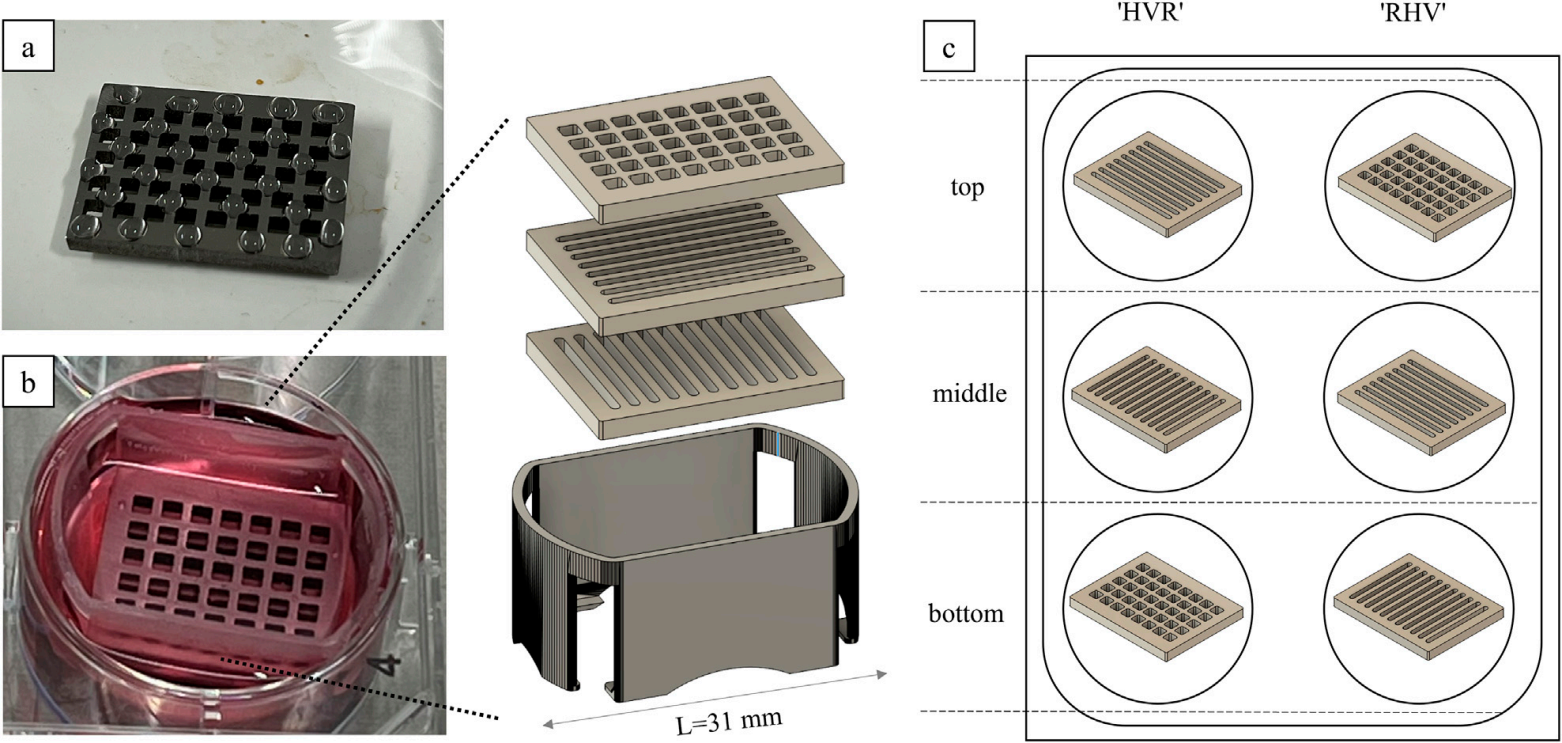
Cell colonization of the Ti alloy sheets. **(a)** The cell suspension is randomly pipetted onto the surface in droplets. **(b)** The platelets are stacked in a self-designed 3D-printed holder and cultivated in a six-well plate. **(c)** The combinations indicate the order of the platelets in the stack from top to bottom.

Primary fibroblasts were washed three times with Dulbecco’s phosphate-buffered saline (DPBS, Merck D8537-500 ML) and then incubated for 30 min at 37 °C with Calcein AM working solution (4 mL DPBS + 2 µL Calcein AM 4 mM, Biomol Cay14948-1) to enable fluorescent green tracking of viable cells (ThermoFisher, 2020). Calcein AM is a cell-permeable dye that, once enters viable cells, is cleaved by intracellular esterases to release membrane-impermeant calcein. Other fluorescent cell-permeant viability/tracking dyes like CellTracker or CFDA could be considered as well (Beem and Segal, 2013). After the incubation time, Calcein AM solution was removed, and the cells were detached using 0.025% trypsin for 3 min at 37 °C (Murkar et al., 2025). After stopping the enzyme reaction using RPMI 1640 (ThermoFisher, 21875-034) + 20% fetal calf serum (FCS, Bio&Sell FBS.S0615), the cell suspension was centrifuged (5 min, 2,300 rpm, 4 °C) (Hasemann et al., 2021). The fibroblasts were counted with trypan blue using a Neubauer counting chamber. For each top platelet, 1 × 10^5^ cells were resuspended in 150 µL fibroblast medium. The cell suspension was applied in 5-µL droplets on top of each sample, ensuring that the cell suspension is not running off (Figure 1a). The samples were incubated for 24 h in the incubator. On the next day, the cell culture medium of the six-well plates was removed, and the Ti-6Al-4V plates were stacked in one well according to Table 1, using a self-developed 3D-printed platelet holder (Figure 1b). The holder was designed using CAD software and 3D-printed of biocompatible resin Bio-Med Clear (Liqcreate). It ensures that the medium flows under the plates and that they remain stacked on top of each other in the correct position. It is designed to fit into a six-well plate and allow three platelets to be stacked on top of each other. Five milliliters of fresh fibroblast medium was added in each well, and the seeded plates were carefully transferred onto non-seeded plates. Medium exchange was done three times a week. On day 3 and day 14, fluorescence microscopy was performed to evaluate cell migration. Therefore, the platelets were carefully separated each in one well of a six-well plate (Figure 1c). After microscopy of both sides of the Ti alloy sheets, they were stacked in the same way as before. On day 14, 3-(4,5-dimethylthiazol-2yl)-2,5-diphenyltetrazolium bromide (MTT) assay was performed (Hasemann et al., 2021). Formazan crystals are formed by metabolic active cells after a 3-h incubation with 0.5 mg/mL MTT solution (Sigma Aldrich, M2128-250 MG) in PBS- at 37 °C. Subsequently, these crystals were solubilized in isopropanol, resulting in purple color formation. Following a 45-min incubation at room temperature, triplicate samples were taken from each well, and the absorbance was measured using a photometer (Hasemann et al., 2021).

### 2.3 Cleaning and sterilization

After each experiment, the Ti alloy sheets were covered with 0.025% trypsin for 15 min. This step was done to ensure that all cell debris was removed from the surface. The process was facilitated by repeated pipetting and shaking. A higher concentration and incubation time may arise for different cells. Subsequently, the Ti alloy plates were rinsed with water, and slits were carefully cleaned with a brush, following overnight incubation in 70% ethanol. The platelets were rinsed again with water, dried at 60 °C and placed in glass Petri dishes. These were heat-sterilized at 160 °C in a UF110 oven (Memmert GmbH) for 6.5 hours. The samples were reused for the next replicate.

### 2.4 Image processing

Fluorescence microscopy images were analyzed using the FIJI/ImageJ software. The removal of the green background fluorescence from each image was achieved through the implementation of background subtraction, utilizing the designated “Subtract Background” function. The images were converted into binary masks by means of the “Make Binary” function. The “Watershed” algorithm was applied to separate the clustered cells due to overlapping fluorescence intensity. The “Analyze Particles” function was utilized to facilitate automatic cell counting, which was subsequently displayed in the program output.

### 2.5 Statistical analysis

All values are expressed in average ±standard deviation if not otherwise stated. Statistical analysis was performed using the Software OriginPro 2023b. Differences between the stacked models and the varying surface conditions were evaluated with the nonparametric Mann–Whitney-U-test, considering p-values <0.05 as significant and p > 0.05 as nonsignificant.

## 3 Results

### 3.1 Characterization of the surface structure

For validation of the surface roughness of the platelets, a confocal microscope (NanoFocus μSurf) was used. With this, several 3D surface parameters, including the arithmetic average height (Sa, Ra), the maximum height (Sz, Rz), and the maximum recess height (Sv, Rv), were measured (Table 2) and are comparable to previous investigations of Regenberg et al. (Sa = 0.067 μm, Sz = 1.5 μm, and Sv = 0.65 μm) (Regenberg et al., 2021). The measurement points of each sample were randomly taken in the center and at the edges of the long and short sides of the platelets.

**TABLE 2:** Surface parameters: arithmetic average height (Sa, Ra), maximum height (Sz, Rz), and maximum recess height (Sv, Rv) of the ground and polished platelets.

Sample		Sa, µm	Sz, µm	Sv, μm	Ra, μm	Rz, μm	Rv, μm
Ground	R	0.066 ± 0.004	1.76 ± 0.17	0.82 ± 0.03	0.044 ± 0.004	0.276 ± 0.022	0.143 ± 0.006
	H	0.084 ± 0.007	2.07 ± 0.58	0.77 ± 0.15	0.071 ± 0.010	0.397 ± 0.045	0.225 ± 0.023
	V	0.082 ± 0.025	1.70 ± 0.23	0.82 ± 0.15	0.058 ± 0.028	0.340 ± 0.149	0.202 ± 0.097
	Mean	0.077 ± 0.017	1.84 ± 0.41	0.80 ± 0.12	0.058 ± 0.011	0.338 ± 0.050	0.191 ± 0.035
Polished	R	0.075 ± 0.015	2.11 ± 0.23	0.99 ± 0.07	0.053 ± 0.016	0.301 ± 0.088	0.173 ± 0.057
	H	0.066 ± 0.007	1.49 ± 0.10	0.77 ± 0.06	0.051 ± 0.006	0.274 ± 0.033	0.149 ± 0.023
	V	0.077 ± 0.008	1.24 ± 0.05	0.69 ± 0.06	0.059 ± 0.015	0.319 ± 0.075	0.190 ± 0.045
	Mean	0.073 ± 0.012	1.62 ± 0.39	0.82 ± 0.14	0.055 ± 0.003	0.298 ± 0.019	0.171 ± 0.016

**FIGURE 2:**
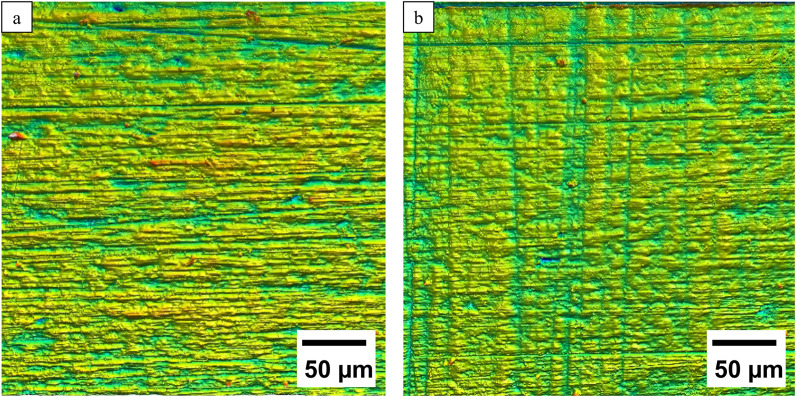
Comparison of representative 3D confocal microscopic images of the **(a)** ground and **(b)** polished surface structures.

The p-values (p > 0.05) demonstrate that there is no significant difference between the roughness values of both surface conditions: p_Sa = 0.8, p_Sz = 0.25, p_Sv = 0.93, p_Ra = 0.67, p_Rz = 0.42, p_Rv = 0.42. However, manual polishing results in a smoother surface, as visualized in the confocal microscope in Figure 2.

### 3.2 Cell migration

**FIGURE 3:**
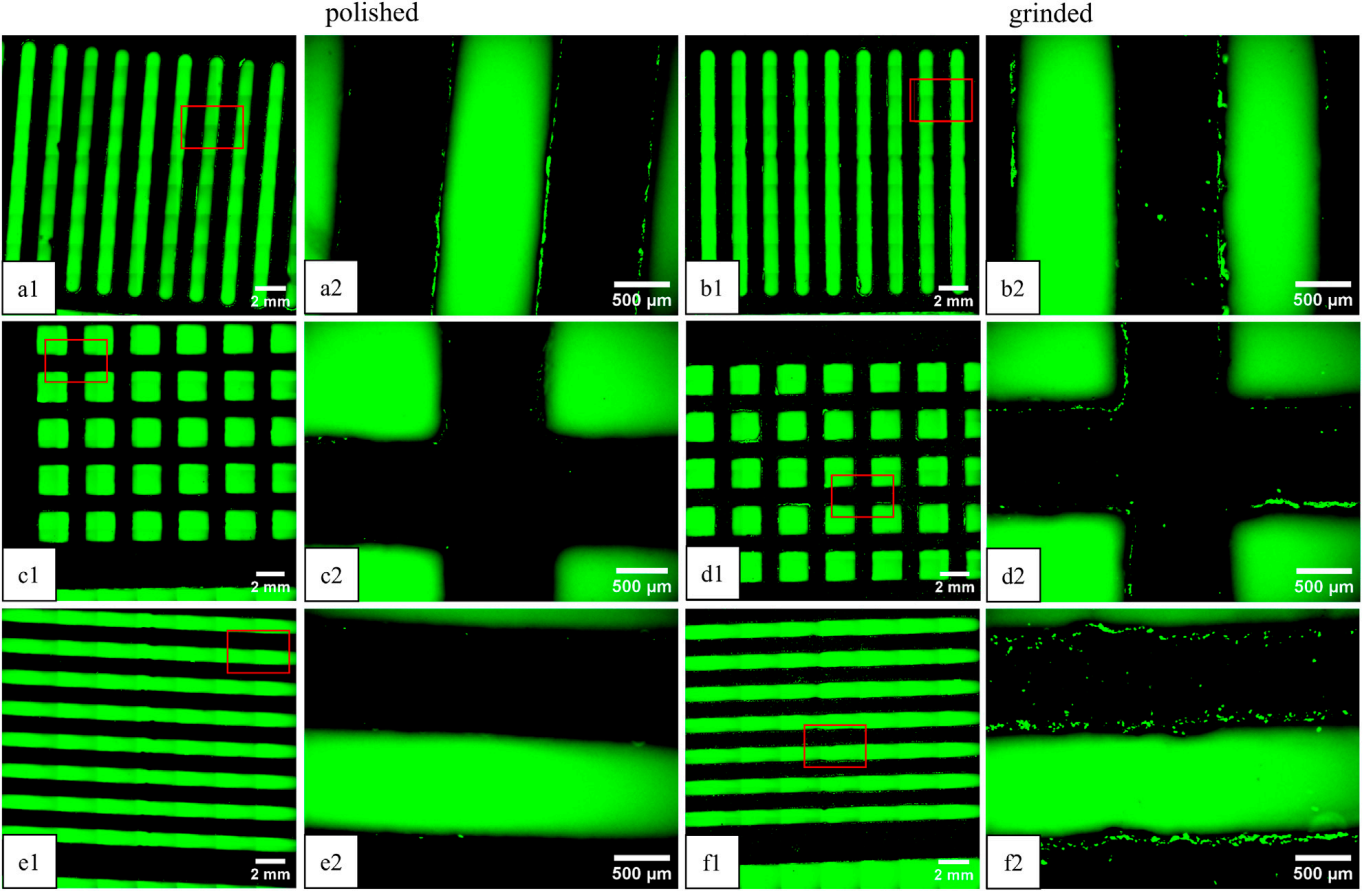
Fluorescence microscopy images of green-labeled fibroblasts on Ti-6Al-4V platelets on day 14 (view of the underside). The plates were stacked in “vertical” on top **(a1, a2, b1, b2)** “rectangular” **(c1, c2, d1, d2)** in the middle, and “horizontal” **(e1, e2, f1, f2)** alignment on the bottom. Comparison of polished (left) and ground plates (right). The images on the right (“x1”) correspond to an enlarged section (4x) of the overview image on the left (“x2”).

Cell attachment and migration of the fluorescent green-labeled fibroblasts on the Ti-6Al-4V platelets were observed on day 3 and day 14. A representative example of fluorescence microscopy images on day 14 with the combination “VRH” is shown in Figure 3.

The location of the cells is in the intermediate spaces between the patterns and on the outside of the platelets. The following cell counts and covered areas were obtained from image analysis of the images shown: V_polished_ = 155 (0.8%), R_polished_ = 46 (0.05%), H_polished_ = 15 (0.02%), V_grinded_ = 136 (0.8%), R_grinded_ = 132 (0.7%), and H_grinded_ = 306 (2%). Additionally, cells are present on all the platelets, including those that were further down the stack. On the upper side of the top platelet, cell clusters have formed, where the cell suspension was applied in droplets on the first day (not shown).

**FIGURE 4:**
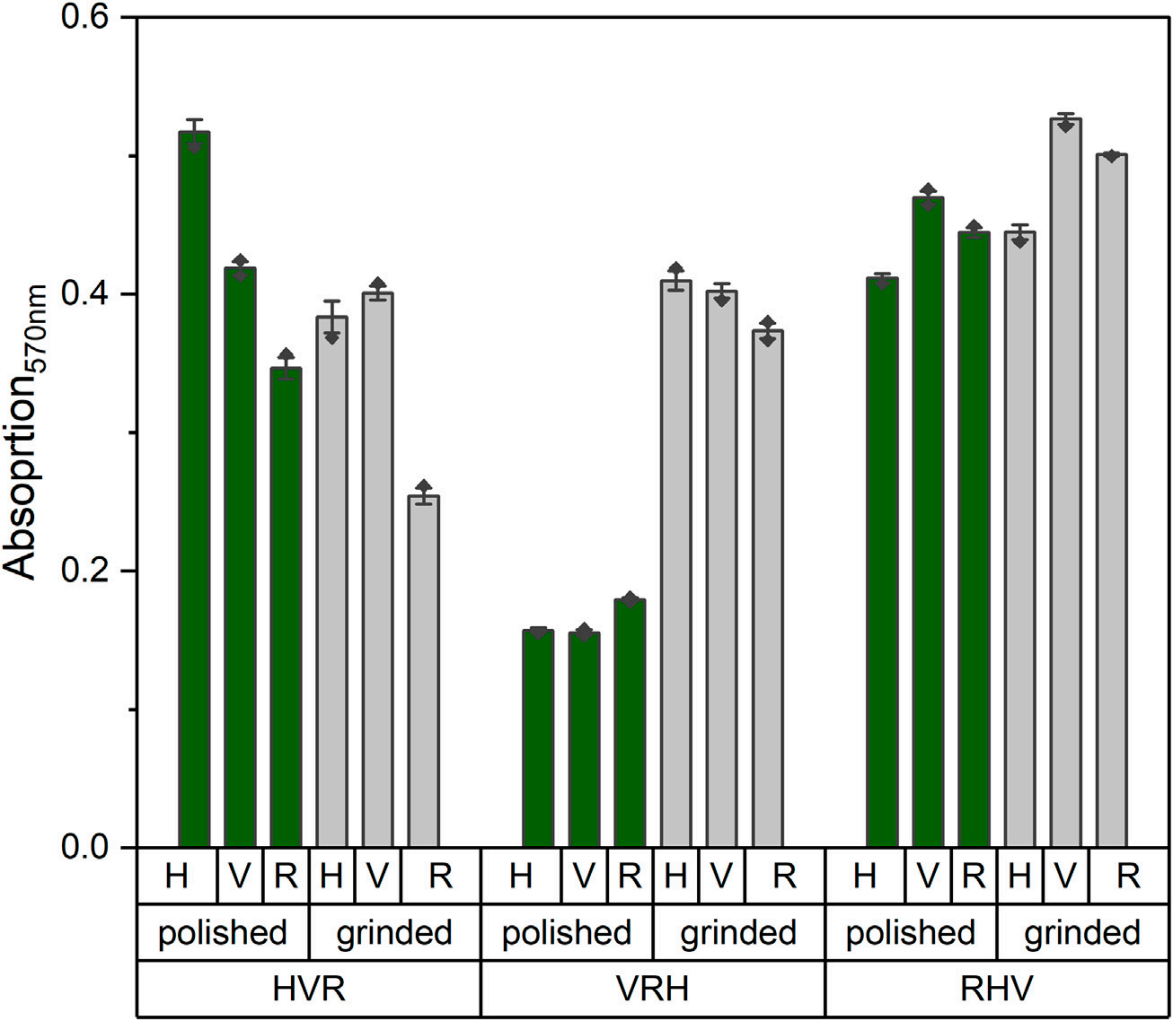
Absorption values of MTT measurements at 570 nm wavelength on day 14 of the stacked models in polished (green) and ground (gray) state. A higher absorption value corresponds to a higher cellular activity.

On day 14, an MTT test was performed to quantify the cellular activity (Figure 4). Absorption values exhibited no consistent differences between the stacked models, and no clear trend was observed between the ground and polished states.

## 4 Discussion

Changes in the porosity and surface properties of metals could be used to optimize implants to reduce rejection reactions and loosening of implants, for instance (Bandyopadhyay et al., 2010). In the present study, a stacked system of Ti-6Al-4V platelets with different patterns was used to produce porous 3D models with varying geometry sequences. By stacking the platelets, cell migration of primary fibroblasts in the individual layers was observed.

Previous studies have investigated the migration of immortalized rat fibroblasts in porous structures of Ti-6Al-4V (Crackau et al., 2020). However, the present study utilized primary human fibroblasts to be physiologically closer to the intended field of application. Fibroblast cells are responsible for producing collagen to replace the extracellular matrix of the injured tissue and are therefore important in wound healing (Kastellorizios et al., 2015). They play a crucial role in the foreign body reaction, which can lead to encapsulation, functional limitations of the implants, or implant loosening (Welch et al., 2020; He et al., 2017). To model early-stage tissue integration, we focused on the migration of primary human fibroblasts into a structured scaffold. *In vivo*, fibroblasts play a key role in the remodeling and stabilization of implants by migrating into the material and depositing the extracellular matrix. The use of primary cells increased the translational relevance of the results, particularly with regard to biocompatibility and scaffold integration.

The observed cell behavior is influenced not only by the choice of cell type but also by the underlying scaffold architecture. Previous studies chose randomized porous structures, resembling a foam, which required estimation of the pore size (Crackau et al., 2020). In contrast, we have used organized structures, thus allowing the calculation of pore size with greater precision. It enables a more comprehensive understanding of the pore geometry and interconnectivity within the 3D scaffold architecture and thus creates a reproducible microenvironment.

The migration of the fibroblasts in depth in all models can be visualized through fluorescence microscopy. Interestingly cell islands are visible on the surface of the topmost platelet, indicating that they correspond to the positions of the droplets of the cell suspension during colonization. This suggests that rather than spreading on the surface of the platelet, the cells migrate directly into the deep, even if there is enough space left in 2D. This behavior can be observed on all surfaces.

Nevertheless, using the MTT assay, it can be shown that the cells grew equally well on the different stacked models. Normalization of the MTT results to the surface area was not necessary as the surface area available for cell attachment on all platelets is approximately 1,500 mm^2^. Furthermore, it is suggested that no substantial differences are visible since the pore channel of the stacked platelets is always similar, resulting in comparable migration paths for the cells. The stacking sequence of the platelets remains the same, but the layer of colonization varies. This layer is important since the pattern may affect the adhesion of the cell suspension to the surface, resulting in variations in the cell count on the platelet. Rectangular platelets have wider bars than vertical and horizontal structures, leading to greater adhesion of the cell suspension to the platelet. Other studies have reported the problem that the cell suspension is running through the pores of the plates during the initial cell seeding (Noskovicova et al., 2021). This may be the source for minor deviations between the models. By applying the cells dropwise on the top platelet, this issue should be prevented in order to create the same starting conditions for all models. The developed holder functioned as a tool to maintain the alignment of the platelets, thereby preventing them from floating away and avoiding direct contact between the platelets and the bottom of the well. The resulting movement of the medium below the model prevented air bubbles from forming inside, which would have negatively affected the oxygen gradient and nutrient supply. Commercial cell culture platforms are available, such as CellCrowns™ (Scaffdex) or Transwell® inserts (Corning), providing a solution to allow medium supply from both the apical and basal sides of cell layers or round scaffolds. However, for rectangular or non-standard scaffold geometries, commercial solutions are limited, making the development of custom-made holders necessary to ensure defined positioning, reproducible experimental conditions, and compatibility with existing laboratory vessels.

**FIGURE 5:**
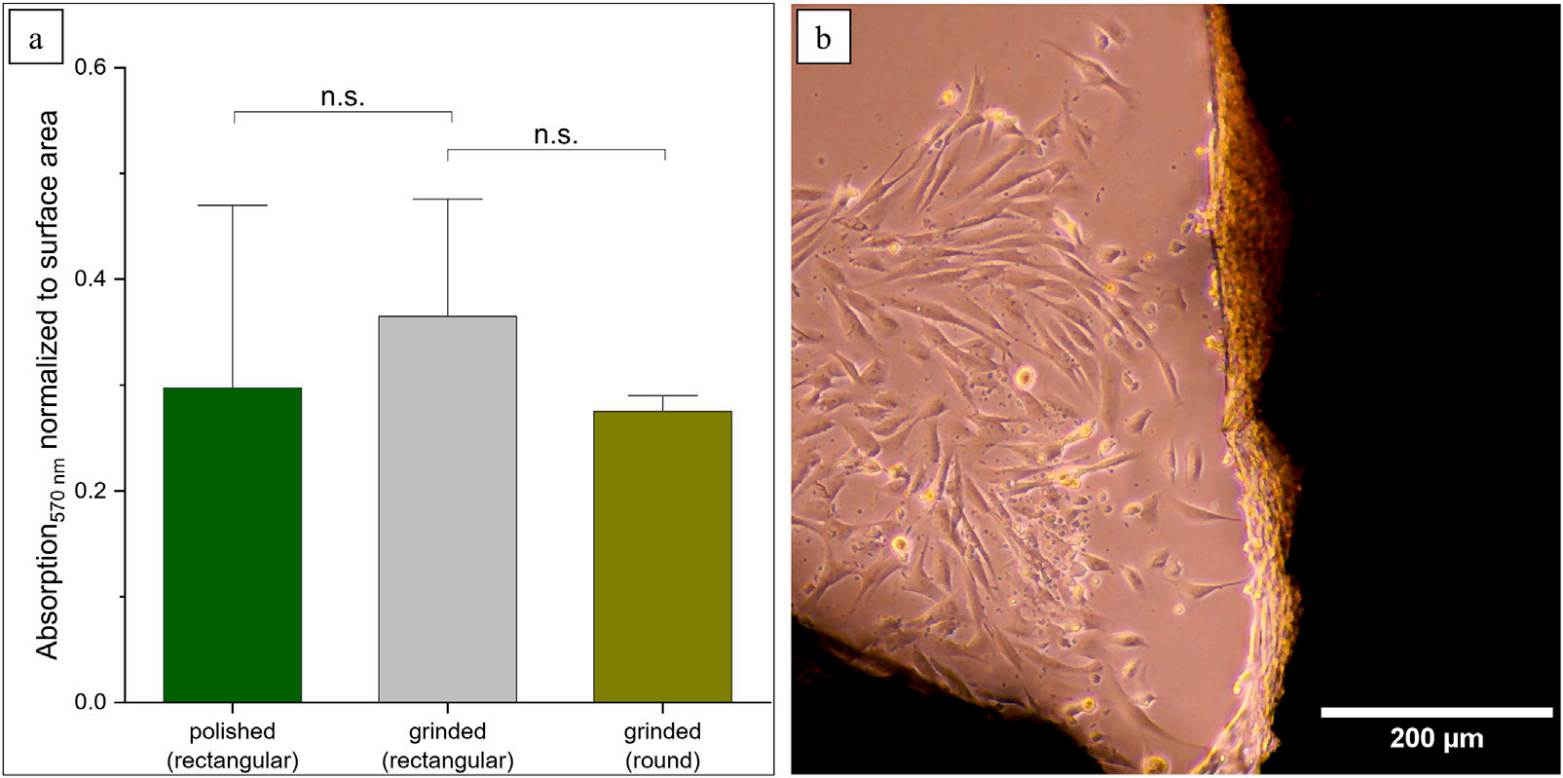
Prospects for more biomimetic structures using round scaffolds. **(a)** MTT results were normalized according to the surface area (n.s. not significant: p_polished_ = 0.60, p_grinded_ = 0.06). **(b)** Formation of cellular sheets in the edges of the structures.

In this model, the process of stacking Ti alloy sheets with different slots resulted in the formation of pore channels measuring up to 1 mm in diameter. In the study conducted by Watanabe et al. (2023), 100-µm-deep groove structures were introduced in Ti-15Mo-5Zr-3Al, and the alignment of osteoblasts within this structure was observed. Since we are in the range of 10-fold higher, smaller structures might be necessary. Therefore, we investigated round Ti-6Al-4V platelets with biomimetic honeycomb structures, which are smaller than the previously described samples (A_round_ = 937 mm^2^). For this reason, the MTT results were normalized to the surface. By calculating the average absorption value of all polished and ground platelets and comparing this with the new samples, no significant differences can be demonstrated (p-value >0.05) (Figure 5a). It could be observed that fibroblasts were forming cellular sheets in the edges of the honeycomb structures (Figure 5b). This corresponds to the expectations according to the literature, where the growth behavior of the cells in curvatures is described (Zadpoor, 2015).

According to the results, it is suggested that cell adhesion and proliferation were equally good on all models. Further research can be conducted on the impact of porosity on cell differentiation. Osteoblasts, which differentiate from stem cells, are suitable for this purpose and should grow into the implant to provide stability (osseointegration) (He et al., 2017). Studies have shown that rough titanium surfaces lead to increased cell attachment and osteoblast differentiation. Ødegaard et al. (2021) found a facilitated differentiation in osteoblasts in scaffolds with pore and lattice diameters of 800 µm. This suggests that further refinement of our porous structures may be necessary.

Although literature suggests that smoother surfaces may promote soft tissue integration (Rabel et al., 2021), this was not reflected in our findings as fibroblast growth showed no significant variation between polished and rough surfaces. A possible explanation for this may lie in the surface characterization results, which revealed only minor differences in topography between the polished and ground surface. One potential area for improvement is the manual surface preparation and treatment. It may be beneficial to automate this process for further investigations. The random sample measurements of the platelets did not reveal any significant differences in the characterized roughness values, which is consistent with the lack of major differences in cell colonization. Therefore, it may be possible to summarize the models without losing any important trends. To further optimize the cell migration behavior, the size of the structures (porous) platelets will be reduced during ongoing experiments using bio-inspired geometries such as honeycomb pores, varying, i.e., the strut number and width, since cells tend to prefer to migrate into the structure rather than cultivate on the respective surface. Nevertheless, as mentioned before, the surface roughness plays an important role during implant ingrowth and will be controlled via automatized metallographic grinding and polishing.

The *in vitro* model presented in this study allows the investigation of material–cell interactions and may support the early screening and optimization of potentially suitable implant surface characteristics. *In vitro* systems are useful for biological evaluation, such as cytotoxicity testing, since they enable fast, cost-effective, and reproducible assessments under controlled conditions. However, *in vitro* assays cannot fully replicate the complex physiological environment encountered *in vivo*. Critical parameters such as vascularization, immune response, mechanical loading and long-term degradation processes remain beyond the scope of cell-based models (Frisch et al., 2023). Consequently, while *in vitro* optimization represents a crucial first step, candidate materials must undergo preclinical *in vivo* evaluation for determining their safety and mechanical stability in a living system. These requirements are consistent with international standards, such as ISO 10993, which outlines the regulatory framework for the biological evaluation of medical devices, including both *in vitro* and *in vivo* endpoints (Frisch et al., 2023). Positioning the present model within this framework highlights its role as a complementary step between experimental testing and clinical use, while not replacing subsequent validation stages.

## 5 Conclusion

Cell migration of fibroblasts was investigated using a stacking model consisting of three differently structured Ti-6Al-4V sheets. By stacking these, pore channels were created, in which cells were observed down to the lowest layer. This suggests the preference of migration in the depth rather than spreading on the surface. The absorption measurements did not follow a consistent trend across the stacked models, and no stable differences were identified between the ground and polished states. This finding suggests that smaller, biomimetic geometries, such as honeycombs, should be used in the future. Since the manual surface preparation revealed no significant differences, an automated process is considered for future evaluations. However, a model for the 3D investigation of biocompatibility and cell migration into a porous implant material was shown under static conditions, which can contribute as a screening tool for early-stage biological evaluation (cytotoxicity). It is hypothesized that the present model can be expanded in perfusion in the future, and the investigation of other cell types, such as osteoblasts, might be of interest.

## Data Availability

The raw data supporting the conclusions of this article will be made available by the authors, without undue reservation.
